# Environmental-friendly regenerated lignocellulose functionalized cotton fabric to prepare multi-functional degradable membrane for efficient oil–water separation and solar seawater desalination

**DOI:** 10.1038/s41598-023-32566-9

**Published:** 2023-03-31

**Authors:** Jiangyi Li, Junkai Gao, Jiangyu Fang, Tian Ling, Mengsheng Xia, Xue Cao, Zhi Han, Yan Chen

**Affiliations:** 1 School of Naval Architecture and Maritime, Zhejiang Ocean University, Zhoushan, 316022 China; 2 Present Address: School of Energy and Power Engineering, Jiangsu University, Zhenjiang, 212013 China

**Keywords:** Environmental sciences, Materials chemistry

## Abstract

Freshwater pollution and shortage have become an imminent problem. Therefore, it is necessary to develop a multi-functional membrane for the production of fresh water. In this work, the regenerated lignocellulose modified cotton fabric was developed as a novel, multi-functional and degradable membrane (LCPT@CF) for efficient oil–water separation and solar steam generation for the first time. The fabrication method has the merits of simple, environmentally friendly and cost effective. The regenerated lignocellulose was adhered on the surface of cotton fabric by tannic acid and polyvinyl alcohol complexes tightly, and the multilayered structures of the LCPT@CF can be formed, which endowed the membranes with underwater superoleophobic property and durability. The underwater superoleophobic property enabled LCPT@CF to purify various kinds of oil-in-water emulsions with a separation efficiency of more than 99.90%. Moreover, benefiting from the excellent photothermal conversion capacity of regenerated lignocellulose, the LCPT@CF achieved high evaporation rate of 1.39 kg m^−2^ h^−1^ and favorable evaporation efficiency of 84% under 1 sun illumination, and the LCPT@CF also presented excellent salt-resistance for evaporating seawater for 20 cycles, without salt accumulation. More importantly, the LCPT@CF could be naturally degradable by microorganisms in the natural condition within 3 months, which had outstanding environmental friendliness. These above results demonstrated that the green and efficient LCPT@CF could play great potential in oil–water separation and sewage purification.

## Introduction

With the increase in population, water shortage has become one of the biggest challenges in the world. Wastewater purification and seawater desalination have become important means to alleviate freshwater shortage^1–3^. Therefore, it is significant to develop low-cost and multifunctional water purification technologies.

In order to solve the problem of water shortage, researchers have devoted themselves to developing various technologies for obtaining freshwater, such as the purification of oil–water mixtures/emulsions and desalination of seawater^4^. Currently, there are many oil–water separation technologies, including oil skimming, centrifugation, air flotation and membrane separation^5^. Among them, membrane separation technology possessed the advantages of highly selectivity, low energy consumption, simple equipment and so on^6^. Meanwhile, membrane separation technology could be used for the purification of oil–water emulsion containing stable surfactants^7,8^. However, the traditional synthesis technologies of membrane still had the disadvantages of complicated preparation, secondary pollution and high-cost expenses of materials^9^. Accordingly, it is imperative to develop membrane materials that are inexpensive, uncomplicated preparation and environmental friendliness for oil–water separation.

As another promising technology for fresh water production, seawater desalination, especially solar steam generation (SSG), had attracted the attention of many researchers due to its environmentally friendly and sustainable characteristics^10–12^. The evaporation efficiency of solar evaporators was determined by many influencing factors, including light absorption, water transportation and thermal management^13^. Among them, light absorption as a crucial part of evaporators was determined by the photothermal materials, which could be divided into metallic materials and carbonaceous materials^14–16^. However, metallic materials were difficult to be used on a large scale due to their high cost^17,18^. On the other hand, some metals with higher plasma frequency occurred plasma resonance only in a specific solar spectrum^19^.

Lignocellulosic was the most abundant renewable resource on earth^20^, but its utilization was not wide^21,22^. Recently, Xia et al. reported an in-situ lignin regeneration approach to prepare regenerated lignocellulosic directly from wood powder^23^. The regenerated lignocellulose inherited the hydrophobic and oleophilic properties of the original lignin, which possessed underwater superoleophobic and underoil hydrophobic properties. These advantages make it possible to use regenerated lignocellulose for oil–water separation. In addition, Zhao et al. demonstrated that lignin nanoparticles can carry out solar-to-thermal conversion^24^. Therefore, the regenerated lignocellulose had significant advantages in both oil–water separation and seawater desalination. As a naturally biodegradable material, the regenerated lignocellulose was a renewable and cost-effective resource that can effectively reduce membrane preparation costs for desalination and oil–water separation. However, to the best of our knowledge, it has not been reported for the regenerated lignocellulosic applied in oil/water separation and solar steam generation.

In this work, by filtering regenerated lignocellulose onto cotton fabric, the lignocellulose modified cotton fabric membrane (LC@CF) was prepared and used for oil/water separation and solar steam generation. However, the regenerated lignocellulose on the surface of cotton fabric could be easily destroyed, thereby reducing the efficiency of oil–water separation and seawater desalination. To solve this problem, a supramolecular adhesive (PT) was used to improve the bond between the regenerated lignocellulose and cotton fabric. Lignocellulose-PVA-TA@cotton fabric membrane (LCPT@CF) was prepared by supramolecular adhesive (PT) to improve the bonding between the regenerated lignocellulose and cotton fabric. When PVA and TA were contacted, the PT complexes with strong adhesiveness were generated owing to the strong hydrogen bonding^25^, which could attach on the cotton fabric and firmly bond the regenerated lignocellulose, thus greatly improving the durability of the membrane.

Herein, we reported a simple and efficient strategy for preparing a regenerated lignocellulosic modified cotton fabric for the first time, and successfully prepared lignocellulose-PVA-TA@cotton fabric membranes (LCPT@CF) possessed underwater superoleophobicity and under-oil hydrophobicity, which could be used for oil–water separation and seawater desalination. Benefiting from the surface wettability of the membrane, the LCPT@CF exhibited excellent performance for the oil-in-water emulsion separation. Furthermore, taking the advantage of the excellent light absorption capacity and hydrophobicity property of lignocellulose, the LCPT@CF possessed favorable desalination property. Moreover, it is exciting that the LCPT@CF could be decomposed by nature within three months. The study focused on the regeneration mechanism of lignocellulose and its role in oil–water separation and seawater desalination when combined with PT complexes. Meanwhile, through the preparation of LCPT@CF, the comprehensive comparison was made against cotton fabrics and LC@CF in terms of surface morphology, chemical composition, surface wettability, and performance in oil–water separation and seawater desalination. The results fully demonstrated that the multifunctional, green and inexpensive membrane has great application prospects in the fields of oil–water separation and seawater desalination.

## Experimental

### Materials

Balsa wood powder sieved below 100-mesh is selected as the raw material. Choline chloride (C_5_H_14_ClNO, > 98%), oxalic acid dihydrate (H_2_C_2_O_4_ 2H_2_O, > 99%), Poly (vinyl alcohol) (PVA, 99%), methyl blue (MB, > 96%), tannic acid (TA, AR) and Sudan III (BS) were provided by Sigma–Aldrich Bio-Chem Technology Co., LTD. Anhydrous ethanol (AR) was purchased from Sinopharm Chemical Reagent Co., LTD. Daily materials such as cotton fibric (CF), lubricating oil, diesel oil, soybean oil, and olive oil were all purchased in local market.

### Preparation of regenerated lignocellulose

Firstly, choline chloride (ChCl) and oxalic acid mixed at a molar ratio of 1:1 were heated to 80 °C for preparation of deep eutectic solvent (DES). Secondly, the cooled DES solution was mixed with balsa wood powder at a ratio of 12:1 by mass, which was then heated at 110 °C for 8 h. Finally, distilled water was added into the resulting brown liquid (50:1 ml/g) and agitated for 2 h to obtain regenerated lignocellulose solution.

### Preparation of LC@CF and LCPT@CF

For the preparation of TA solution, 2 g TA was added into 100 ml distilled water and stirred for 5 min at room temperature. In terms of PVA solution, 5 g PVA was added into 100 ml distilled water, which was then stirred for 30 min at 95 °C.

First of all, the cotton fabric was immersed in ethanol, which was then cleaned by ultrasonic wave for 10 min. After rinsing with distilled water, the treated cotton fabric was dried under normal temperature, which was represented as Pre-CF. Then 25 ml of regenerated lignocellulose solution was filtered onto the Pre-CF surface to get the LC@CF.

Additionally, Pre-CF was immersed in TA solution for 30 min (marked as TA@CF). Then 25 ml of the regenerated lignocellulose solution and 5 ml PVA solution was mixed and stirred for 5 min. Finally, the mixture was filtered onto the TA@CF surface to get the LCPT@CF.

### Separation of emulsions and oil–water mixtures

The surfactant-stabilized oil-in-water emulsion can be obtained by mixing CTAB, water and oil with stirring. 0.6 g oil (such as olive oil, diesel oil, soybean oil, lubricating oil) and 0.03 g CTAB were added into 300 ml distilled water, which was then stirred at 2000 rpm/min for 2 h to prepare oil-in-water emulsions. Oil–water mixtures were prepared by mixing water and oil at a volume ratio of 1:1.

In order to detect the reusability of the membrane, multiple cyclic separation experiments were carried out. In each cycle, 25 ml of oil-in-water emulsions or 50 ml of oil–water mixtures were used. The separation efficiency (R) and membrane flux (J) were calculated as follow^26^:

1 $$R = \left( {1 - \frac{{C_{p} }}{{C_{f} }}} \right) \times 100\%$$

where $C_{p}$ and $C_{f}$ represent the oil concentration in the emulsions and filtrates, respectively.

2 $$J = \frac{V}{A \cdot \Delta t \cdot P}$$

where J (Lm^−2^ h^−1^ bar^−1^) is the average membrane flux, Δt (h) represents the separation time, V (L) represents the filtered volume, A (m^2^) represents the effective filter material area, which was 12.56 × 10^–4^ m^2^, and P (bar) represents the vacuum pump pressure, which was 0.9 bar.

### Anti-oil fouling tests

The flux recovery rate (FRR), reversible fouling ratio ($R_{r}$) and irreversible fouling ratio ($R_{ir}$) were used to calculate the anti-oil pollution performance of LCPT@CF membranes. FRR, RR and RIR were obtained by the following Eqs. (3), (4) and (5), respectively.

3 $$FRR = \left( {\frac{{J_{w,b} }}{{J_{w,a} }}} \right) \times 100\%$$

4 $$R_{r} = \left( {\frac{{J_{w,b} - J_{p} }}{{J_{w,a} }}} \right) \times 100\%$$

5 $$R_{ir} = \left( {\frac{{J_{w,a} - J_{w,b} }}{{J_{w,a} }}} \right) \times 100\%$$

where $J_{w,a}$ (Lm^−2^ h^−1^ bar^−1^) is the initial pure water flux of LCPT@CF, $J_{w,b}$ (Lm^−2^ h^−1^ bar^−1^) is the pure water flux of cleaned LCPT@CF after cycle emulsions filtration, $J_{p}$ (Lm^−2^ h^−1^ bar^−1^) is membrane flux of emulsion separated in the last cycle of LCPT@CF.

### Solar desalination tests

The samples were put on the sponge and were floated in the beaker which was filled with 50 ml of distilled water. A xenon lamp (PLS-SXE300, Beijing Perfectlight) with an AM1.5 optical filter was used to simulate sunlight. An optical power meter (PL-MW2000, Beijing Perfectlight) was employed to adjust the intensity of light. Meanwhile, the mass changes and surface temperatures of SSG system were real-timely recorded via an electronic microbalance (0.001 g in accuracy) and an infrared camera (FLUKE TiS20+), respectively. The ambient temperature and relative humidity during evaporation test were maintained at 25 °C and 50%, respectively.

The evaporation rate ($v$) can be obtained based on Equation:

6 $$v = \frac{m}{A}$$

where $v$ is the evaporation rate (kg m^−2^ h^−1^), $m$ is the mass change of water caused by evaporation (kg), $A$ is the evaporation area of the sample (m^2^).

The evaporation efficiency (*η*) was calculated using the following formula^27^:

7 $$\eta = \frac{{\Delta v\left( {C \times \Delta T + H_{v} } \right)}}{I} \times 100\%$$

where $\Delta v$ is the net evaporation rate (kg m^−2^ h^−1^), $C$ is the specific heat volume of water (4.2 J g^−1^ K^−1^), $\Delta T$ is the elevated temperature during evaporation (°C), $H_{v}$ is the latent heat of water evaporation (2256.0 kJ kg^−1^), $I$ is the power density of solar illumination (1 kW m^−2^).

### Characterization

The morphology of CF, LC@CF and LCPT@CF membranes was observed by scanning electron microscope (SEM, FEG250, Quanta, USA). Infrared spectra were recorded by reflectance-Fourier transform infrared spectroscopy (ART-FTIR, VECTOR22, Bruker, Germany). The functional groups of CF, LC@CF and LCPT@CF membranes were measured by X-ray photoelectron spectroscopy (XPS) spec-trometer (Thermo VG, ESCALAB250, USA). The water contact angle (WCA) in air, underwater oil contact angle (UOCA), and under-oil water contact angle (UWCA) were obtained by JY-82 contact angle meter. The oil content analyzer (JC-OIL-8, Qingdao Juchuang Instruments Co., Ltd.) was used to check the purity of the separated water, and the biological microscope (CX41-DP27, Shanghai Koumi Instruments Co., Ltd., China) was used to observe the microscope images of the emulsions.

## Results and discussion

### Synthesis mechanism of LCPT@CF

**Figure 1:**
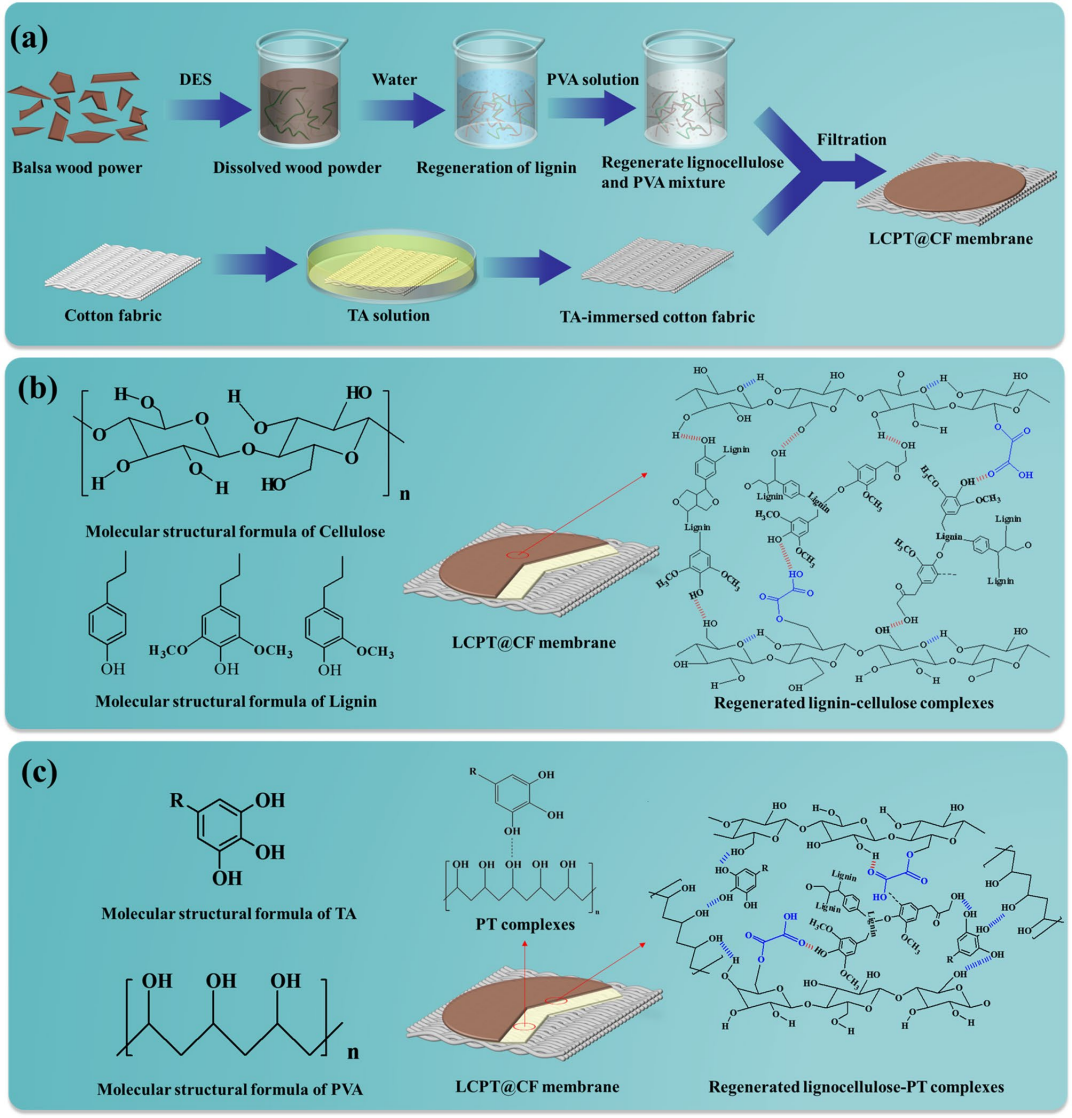
(**a**) Synthesis path of the LCPT@CF. (**b**) Schematic diagram of the dual applications of the LCPT@CF and the interactions of regenerated lignin-cellulose complexes and PT complexes.

The preparation procedures of the LCPT@CF were divided into two steps, which were shown in Fig. 1a. Step 1, the powder of balsa wood was dissolved with a kind of green and degradable deep eutectic solvent (DES). Then, water was poured into the mixed DES solution, and due to the hydrophobicity of native lignin, the dissolved lignin would be regenerated from DES solution^28^. Finally, the regenerated lignocellulose solution and PVA solution were mixed. Step 2, the cotton fabric was immersed in TA solution for 30 min, and then the regenerated lignocellulose and PVA mixture was filtered onto the TA-immersed cotton fabric to get the LCPT@CF.

DES was composed of choline chloride (ChCl) and oxalic acid^29–31^. The strong hydrogen bond (OH-Cl) formed by oxalic acid and ChCl could not only keep DES in a stable liquid state (Fig. S1), but also could break the hydrogen bonds between the cellulose fibers to break down micro and nano fibers and dissolve the lignin. In addition, the hydrogen bond formed by oxalic acid and ChCl could help protons dissociate in acid, which could increase the acidity of DES^30,32^. These processes could further enhance the dissolution speed of wood powder.

In Step 1, the regenerated lignin was cross-linked with micro-nanofibers of cellulose through hydrogen bonding to form regenerated lignocellulose. Collaboration between cellulose and lignin of regenerated lignocellulose was essential for the performance of LCPT@CF^33,34^. The interaction between the regenerated lignin and the cellulose micro/nano fibers was illustrated in Fig. 1b. It is clear that the regenerated lignin closely collaborated with the cellulose micro/nano fibers comprising hydroxyl and oxalic acid-induced carbonyl groups by van der Waals forces (COO–HO) and hydrogen bonds (OH–HO), which could form stable regenerated lignin-cellulose supramolecular complexes^23^. As a result, LCPT@CF has a high stability.

In Step 2, during the filtration of the regenerated lignocellulose and PVA mixture into TA-submerged cotton fabric, PVA can cross-link with TA through hydrogen bonding to form PT complexes with high adhesion^35,36^. As shown in Fig. 1c, the layer of PT complexes can be formed between the regenerated lignocellulose layer and the cotton fabric. Meanwhile, the hydroxyl groups of the cellulose micro/nano fibers and lignin in the regenerated lignocellulose could also connect with the PT complexes by hydrogen bonds, and the chemical molecular formulae of PT complexes and lignocellulose-PT were shown in Fig. 1c. These processes could not only further strengthen the bonding between the regenerated lignocellulose and cotton fabric, but also improve the overall durability and mechanical strength of the LCPT@CF.

### Characterization of the membranes

#### Surface morphology of the membranes

**Figure 2:**
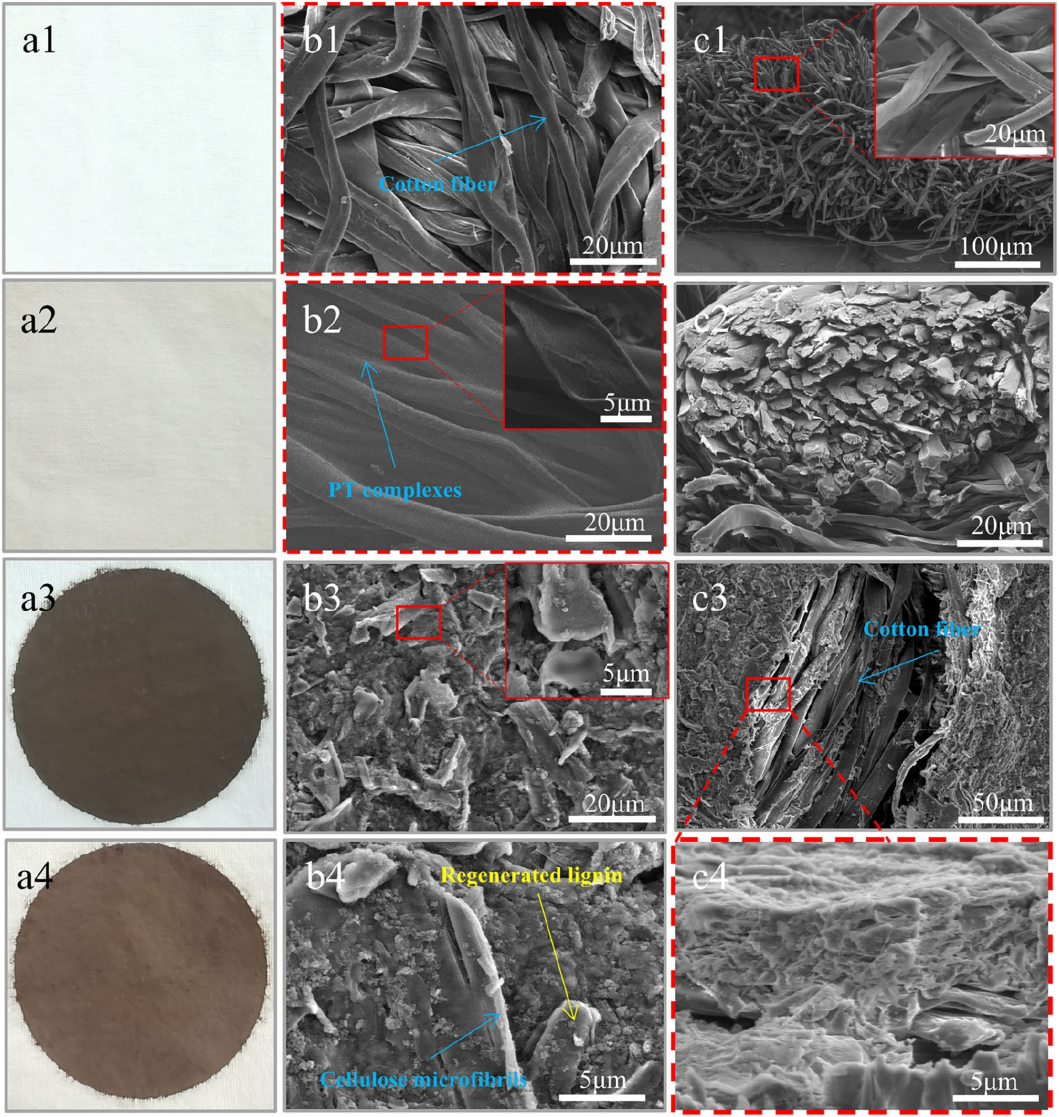
(**a1**–**a4**) Photograph of original CF, PT@CF, LC@CF and LCPT@CF membranes. (**b1**–**b4**) Top-view SEM images of original CF, PT@CF, LC@CF and LCPT@CF membranes. (**c1**–**c4**) Cross-sectional SEM images of original CF, PT@CF, LC@CF and LCPT@CF membranes.

The morphologies of the membranes before and after modification were analyzed by SEM in Fig. 2. The kind of cotton fabric had an EPI (Ends per inch) of 68/inch, a PPI (Picks per inch) of 42/inch, a yarn count of 40 s, and a GSM (Grams per square meter) of 126 g/m^2^. And the cotton fabric was cut into 5 cm × 5 cm wide squares for testing. As illustrated in Fig. 2a1–c1, the original cotton fabric was white and soft, and the morphologies of the cotton fibers appeared smooth and flexible. The cotton fabric turned to be yellowish after the modification of PVA and TA, and the cotton fibers became stiff in texture (Fig. 2a2–c2). Meanwhile, as exhibited in Fig. 2b2 and c2, the cross-link of PT between the cotton fibers can be obviously observed, which could not only strengthen the integrity of the cotton fibers, but also improve the durability of cotton fabric. Furthermore, the holes of cotton fabric were densified after the regenerated lignocellulose was filtrated on its surface (Fig. 2a3,a4). Therefore, both the LC@CF and LCPT@CF membranes showed a uniform and dense surface with high roughness.

As observed from Fig. 2b4, the regenerated lignin, a natural degradable binder, could tightly encapsulate the cellulose micro/nano fibers obtained from the defibrillation of wood powder by DES, which could improve the interaction with cellulose micro/nano fibers. As observed in Fig. 2c3 and c4, both the LC@CF and LCPT@CF membranes were consisted of dense layered structures, which were composed of regenerated lignin and cellulose micro/nano fibers. The difference between the LC@CF and LCPT@CF membranes was that the supramolecular adhesive formed by PT complexes was presented between the regenerated lignocellulose layer and the cotton fabric on the LCPT@CF membrane, which further strengthened the bonding between the regenerated lignocellulose and the cotton fabric. As a result, the LCPT@CF exhibited outstanding mechanical properties and durability in the experiment of separation cycles for oil–water emulsions and mixtures.

#### Chemical analyse of the membranes

**Figure 3:**
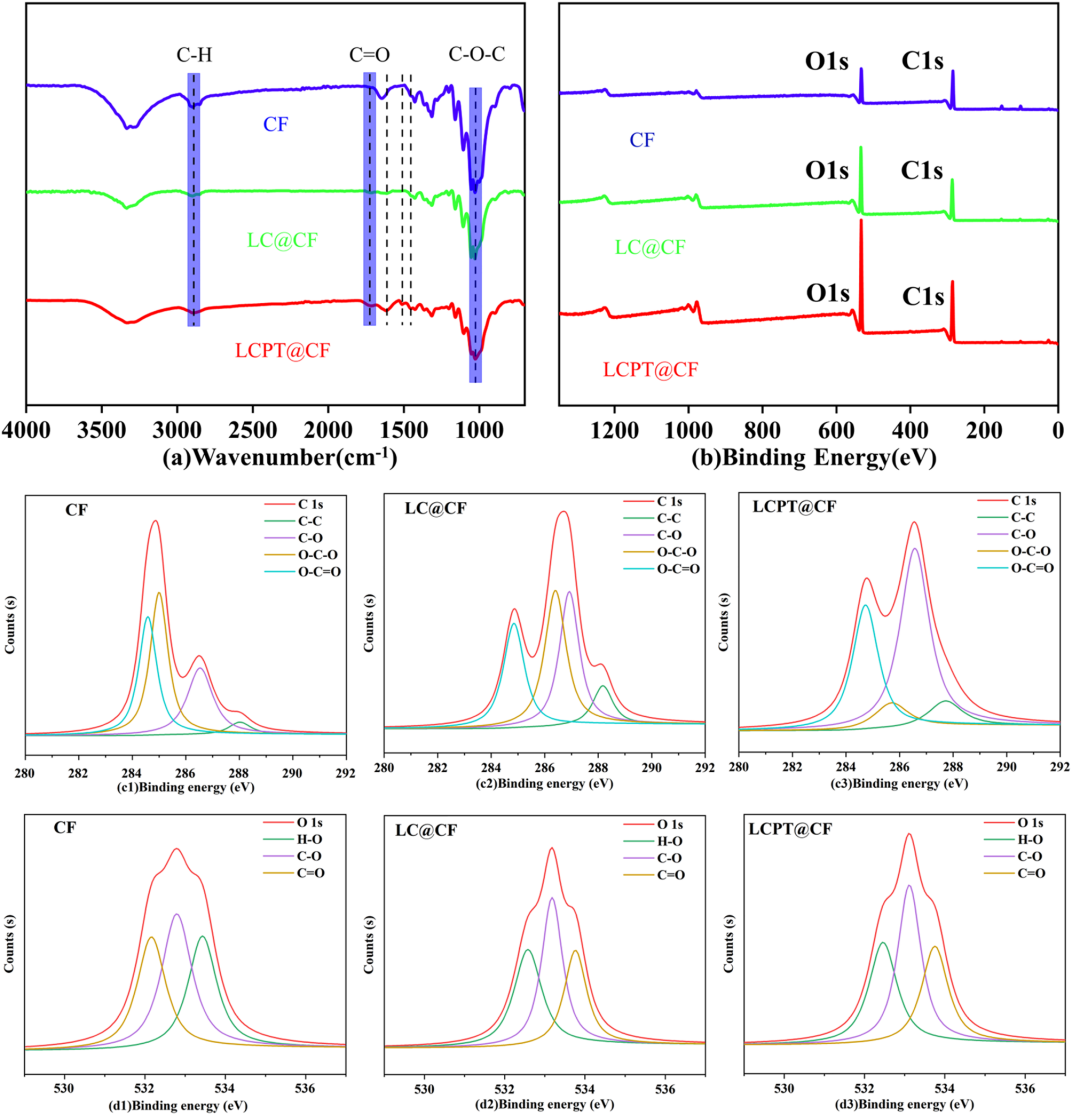
(**a**) The FT-IR spectrum of the membranes. (**b**) The XPS spectrum of the membranes. (**c1**–**c3**) The XPS C1s spectra of original CF, LC@CF and LCPT@CF membranes. (**d1**–**d3**) The XPS O1s spectra of original CF, LC@CF and LCPT@CF membranes.

Fourier transform infrared (FTIR) spectra was conducted to investigate the composition of the original CF, LC@CF and LCPT@CF membranes to further explore the functional groups of the regenerated lignocellulose and PT complexes. As depicted in Fig. 3a, the absorption bands of LC@CF and LCPT@CF membranes were located at 1616 cm^−1^, 1512 cm^−1^ and 1452 cm^−1^, respectively, which were attributed to the aromatic skeleton stretching of lignin ^37^. The results could verify the existence of lignin in the LC@CF and LCPT@CF membranes. Furthermore, the absorption bands at 2893 cm^−1^ and 1053 cm^−1^ represented the vibration of C–H stretching in methylene group (–CH_2_) and C–O–C stretching in cellulose, respectively^38,39^, which indicated that there was a large amount of cellulose on the LC@CF and LCPT@CF membranes. Meanwhile, compared to the LC@CF, the absorption band corresponding to the stretching vibration of carbonyl group (C=O) at 1723 cm^−1^ was detected in the LCPT@CF membrane, ascribing to that the TA deposited on the cotton fabric was cross-linked with PVA to form PT complexes^40,41^.

The chemical components of original CF, LC@CF and LCPT@CF membranes were analyzed by XPS. In Fig. 3b, the modified LC@CF and LCPT@CF membranes exhibited sharp C 1*s* and O 1*s* peaks compared with the original cotton fabric. The original cotton fabric was composed of 74.57% carbon, 25.06% oxygen and 0.37% nitrogen, and the oxygen and carbon content of modified LC@CF and LCPT@CF membranes exhibited a clear ascending tendency (Table S1). In addition, the O/C ratio of the LCPT@CF was significantly different from that of the LC@CF, indicating that there was PVA on the surface of the LCPT@CF.

The above study results indicated that the regenerated lignin can interact with cellulose by hydrogen bonds. In addition, the hydroxyl groups of the cellulose micro/nano fibers and lignin in the regenerated lignocellulose could also connect with PT complexes by hydrogen bonds, and thus the regenerated lignocellulose and the PT complexes were tightly connected, which improved the overall durability and mechanical properties of the LCPT@CF.

### Wettability and anti-fouling properties of the membranes

**Figure 4:**
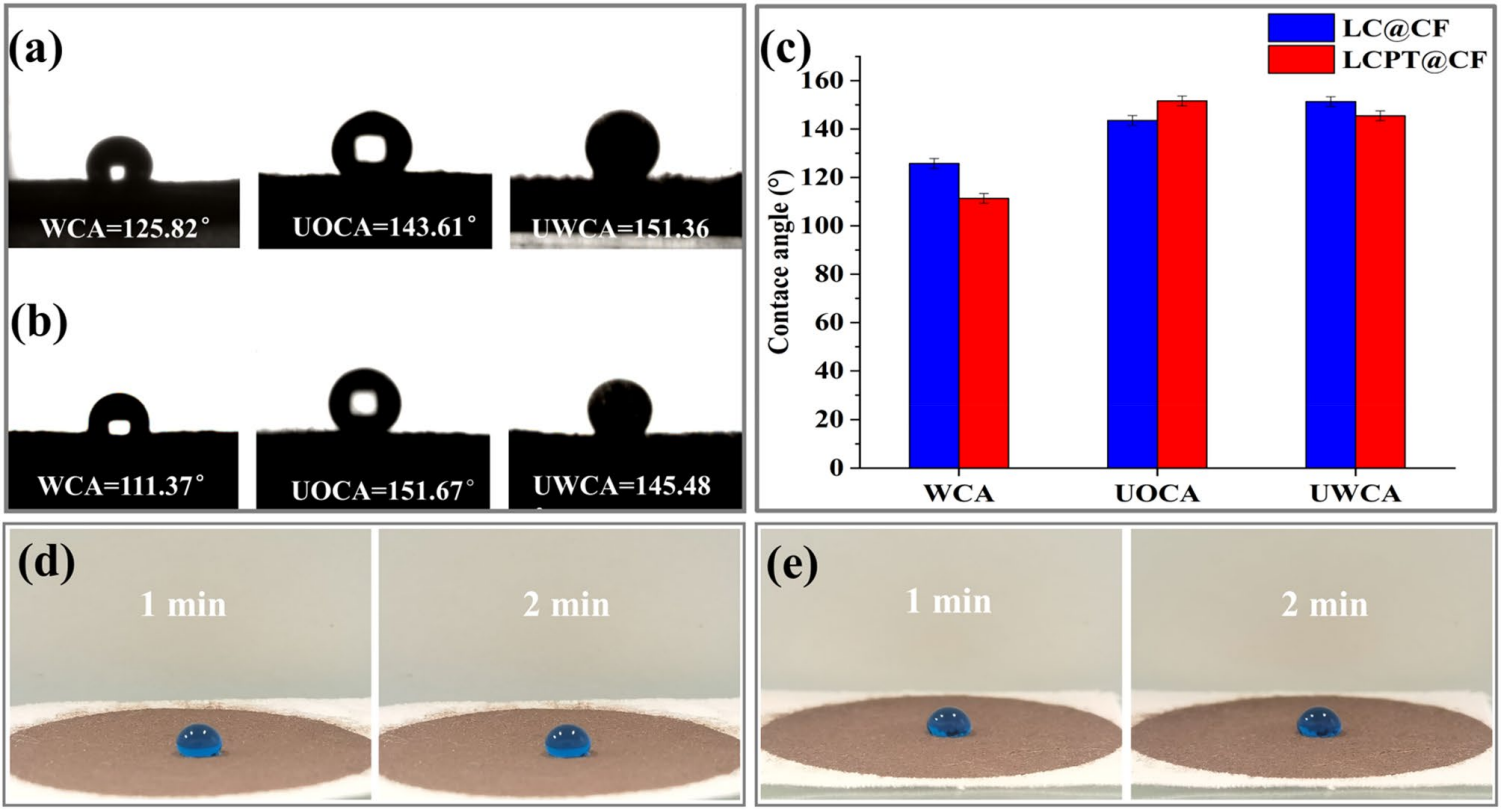
(**a**) Water contact angle (WCA), underwater oil contact angle (UOCA) and under-oil water contact angle (UWCA) of LC@CF. (**b**) Water contact angle (WCA), underwater oil contact angle (UOCA) and under-oil water contact angle (UWCA) of LCPT@CF. (**c**) Comparison of water contact angle (WCA), underwater oil contact angle (UOCA) and under-oil water contact angle (UWCA) between LC@CF and LCPT@CF membranes. (**d**) Water droplet on the LC@CF after 1 min and 2 min. (**e**) Water droplet on the LCPT@CF after 1 min and 2 min.

The wetting performance of membrane was very significant for its application in oil–water separation, thus the wettability of LC@CF and LCPT@CF membranes were investigated by water contact angle (WCA), underwater oil contact angle (UOCA) and under-oil water contact angle (UWCA) experiments. As shown in Fig. 4, the variation of wetting behaviors of LC@CF and LCPT@CF in different environment was investigated. Firstly, the wetting behaviors of LC@CF and LCPT@CF were studied in air condition. The WCA of LC@CF and LCPT@CF were about 125.82° and 111.37°, respectively (Fig. 4a,b), and the water droplets could maintain the initial size after 2 min (Fig. 4d,e), suggesting the inherent hydrophobicity of LC@CF and LCPT@CF, which were ascribed to that there were a mass of hydrophobic alkyl and phenylpropane groups in the molecules of the regenerated lignin^23^. Additionally, it is noteworthy that the hydrophobicity of LCPT@CF declined after it was modified by PT complexes, and the WCA of LCPT@CF decreased to 111.37° (Fig. 4b), which could be attributed to that small amount of hydrophilic PVA were left on the LCPT@CF membrane^42^.

Besides, the wetting behaviors of LC@CF and LCPT@CF membranes were also studied in water condition, and underwater oil contact angle of the membranes was tested by using the insoluble organic solvent of tetrachloroethylene with a higher density than that of water. As exhibited in Fig. 4, the underwater oil contact angles (UOCA) of LC@CF and LCPT@CF membranes reached 143.61° (Fig. 4a) and 151.67° (Fig. 4b), respectively, which showed the underwater oleophobicity of LC@CF and the underwater superoleophobic performance of LCPT@CF. These wetting behaviors were attributed to that the hydration layer was formed by the strong hydrophilic ability of the cellulose on the surface of LC@CF and LCPT@CF membranes, and moreover the hydration layer could prevent the oil droplets from passing through the surface of membranes^43,44^.

Eventually, the wetting behaviors of LC@CF and LCPT@CF membranes were studied in oil (olive oil) condition. As depicted in Fig. 4, the underoil water contact angles (UWCA) of LC@CF and LCPT@CF membranes were 151.36° and 145.48°, respectively, which suggested the underoil superhydrophobicity of the LC@CF and the underoil hydrophobicity of the LCPT@CF. The results showed that the oil layer of repelling water on the surface of LC@CF and LCPT@CF membranes was formed, which was ascribed to that the regenerated lignin in the lignocellulose possessed high affinity to the oil^45^.

Furthermore, based on the comparison of contact angles of the LC@CF and LCPT@CF membranes in Fig. 4c, it can be concluded that the LCPT@CF possessed the underwater superoleophobicity and underoil hydrophobicity. While water contacted with the LCPT@CF, the hydration layer of repelling oil can be formed on the surface of the LCPT@CF, which allowed water to pass through the membrane and prevented oil from passing through it, and thus the demulsification process was achieved. Therefore, the wetting behavior of the LCPT@CF can promote the oil–water separation.

The anti-fouling performance of the membrane is also an important indicator to characterize the property of oil–water separation. In order to investigate the anti-fouling performance of the membrane, the adhesion of oil for the LCPT@CF was tested under water, and the process was shown in (video S1). The anti-fouling performance of the LCPT@CF was detected by the insoluble organic solvent (tetrachloroethylene). It could be seen that the oil droplets (tetrachloroethylene) quickly slipped off without any adhesion after contacting with the LCPT@CF in water, which exhibited that the LCPT@CF possessed high resistance for oil in water.

### The separation properties of the membranes for oil-in-water emulsion

#### Separation properties for oil-in-water emulsion and oil–water mixtures

The separation property of the membrane was affected by its wetting performance. Depending on the wettability of the modified membranes, different types of oil-in-water emulsions could be separated by the modified membranes. Firstly, we prepared the emulsions with five types of oils (lubricating oil, diesel oil, olive oil, cyclohexane and soybean oil), and the particle size and stability of the oil droplets were checked by the biomicroscope. As an example, the emulsion of olive oil remained highly stable within 48 h (Fig. S2).

**Figure 5:**
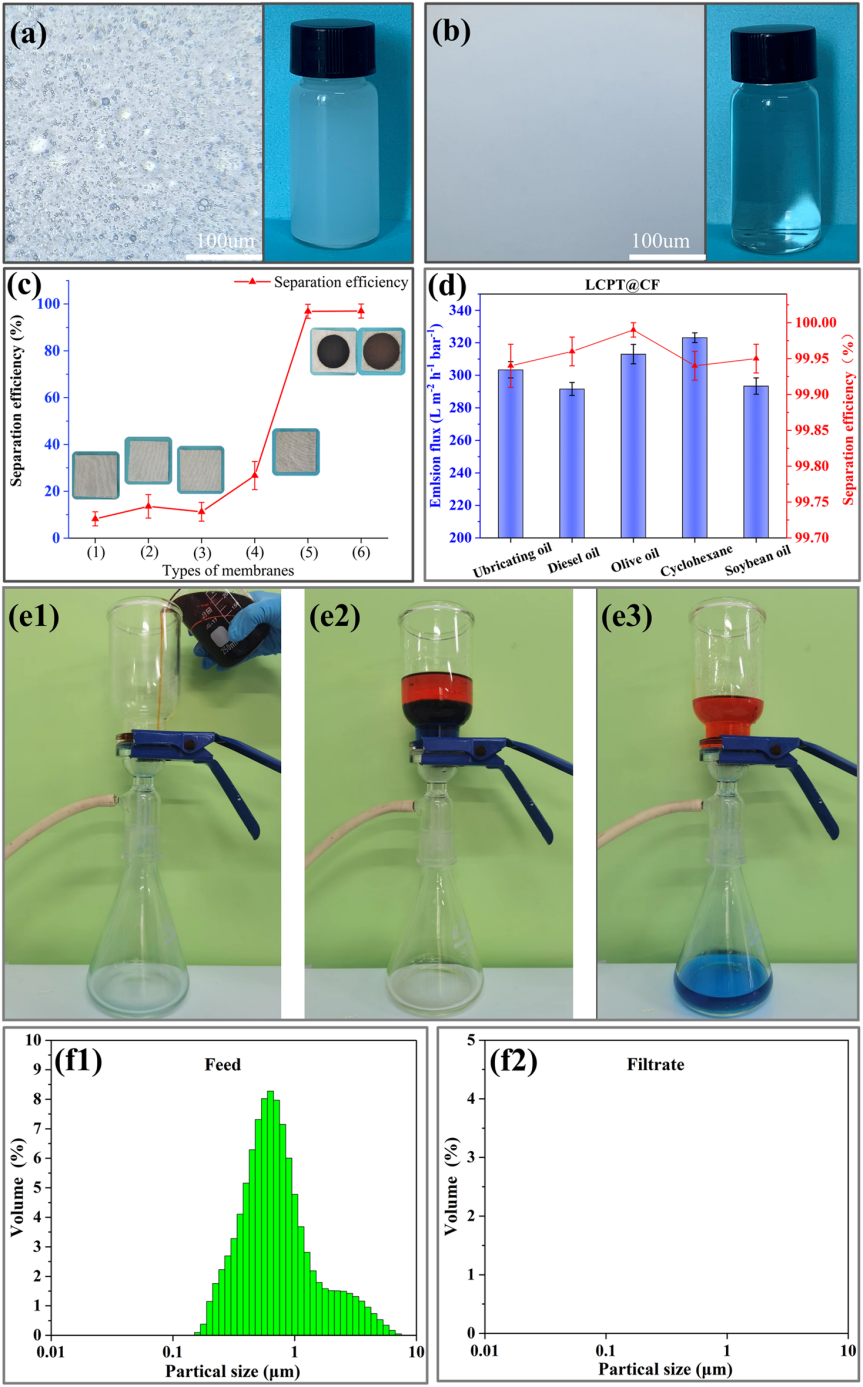
(**a**) Biomicroscope image of olive oil-in-water emulsion. (**b**) Biomicroscope image olive oil-in-water emulsion after filtration by LCPT@CF. (**c**) Separation efficiency and photographs of various membranes ((1) original-CF, (2) TA@CF, (3) PVA@CF, (4) PT@CF, (5) LC@CF, (6) LCPT@CF). (**d**) Separation efficiency and flux of various oil-in-water emulsions by LCPT@CF. (**e1**–**e3**) Photos of oil–water mixtures separation by LCPT@CF. (**f1**–**f2**) Particle size distribution of olive oil-in-water emulsion of feed and filtrate.

The distribution of oil droplets before and after filtration was compared in the surfactant-stabilized oil-in-water emulsions. Before separation in Fig. 5a, there was a wide distribution of oil droplets, and the concentration of the olive oil-in-water emulsion was 2000 ± 200 mg/ml as determined by an infrared oil content analyzer. However, after filtering by LCPT@CF in Fig. 5b, the filtrates of the emulsion were transformed into clear and transparent liquid. The exceptional separation capabilities of the LCPT@CF membrane were demonstrated by the particle size distribution analysis using DLS in Fig. 5f1–f2. Prior to separation, the emulsion displayed a particle size distribution within the range of 0.1–10 μm. After the LCPT@CF separation, no particle size distribution was detected, and the separation efficiencies could reach 99.99%. These results highlighted the effectiveness of the LCPT@CF membrane in separating the olive oil-in-water emulsion and providing a purified end product. The separation processes for the oil-in-water emulsion using LCPT@CF were demonstrated in (video S2), further emphasizing the potential of this membrane for industrial applications.

In addition, the excellent performance of LCPT@CF in separating oil–water mixtures was demonstrated in Fig. 5e1–e3. To evaluate the membrane's separation efficiency, a mixture of 100 g of olive oil stained with Sudan Red III and 100 g of water stained with methyl blue was prepared. The vacuum pump was then initiated, causing the water phase to rapidly penetrate the membrane under a pressure of 0.09 MPa, while the oil phase remained on the upper surface of the LCPT@CF membrane, as shown in Fig. 5e1–e3. The separation efficiencies of LCPT@CF could reach 99.99%, indicating its high capacity to effectively separate oil–water mixtures.

In order to further characterize the separation efficiency of various membranes for emulsions, the separation performance of the original CF, TA@CF, PVA@CF, PT@CF and LC@CF membranes was also tested, and the oil content in the filtrates were measured. The separation efficiencies of the original CF, TA@CF, PVA@CF, PT@CF, and LC@CF membranes were 8.23%, 13.57%, 11.24%, 26.73%, and 99.92%, respectively. According to the results above, it could be turned out that the membranes contained the regenerated lignocellulose was able to separate oil-in-water emulsion effectively (Fig. 5c). The results exhibited that the hydrated layer on the LC@CF membrane surface could be formed by the cellulose micro/nano fibers of hydrophilicity in the regenerated lignocellulose, which could only pass water but not oil, and thus the demulsification process was achieved.

Additionally, the layered structures composed of lignin and cellulose in the regenerated lignocellulose also promoted the oil–water separation of the membrane. As illustrated in Fig. 5d, the separation efficiencies and fluxes of the five kinds of the emulsions (lubricating oil, diesel oil, olive oil, cyclohexane and soybean oil) by the LCPT@CF were also measured. For the five kinds of the emulsions, the membrane fluxes of LCPT@CF were in the range of 292–326 L/m^2^ h·bar, and the separation efficiencies were exceeded 99.90% for lubricant oil-in-water, diesel oil-in-water, olive oil-in-water, cyclohexane-in-water, and soybean oil-in-water emulsion (99.94%, 99.96%, 99.99%, 99.94%, 99.95% respectively), suggesting the outstanding separating ability and wide application performance of LCPT@CF. The separation efficiencies and fluxes were slightly different due to the different viscosities and densities of different types of oils. For the olive oil-in-water, as it can be saw that the flux (328.92 L/m^2^ h·bar) of the LC@CF was higher than that (313.03 L/m^2^ h·bar) of the LCPT@CF, and this result could be caused by the reduction of pore size of the LCPT@CF, which further induced the lower flux but the higher separation efficiency.

The excellent oil–water separation ability of LCPT@CF was attributed to the cooperation of lignin and cellulose in regenerated lignocellulose. Due to the hydrophilicity of cellulose, a hydrated layer could be formed on the LCPT@CF, which allowed water to pass through the membrane and prevented oil from passing through it, thus achieving the purpose of demulsification. Meanwhile, regenerated lignin can wrap micro/nano fibers of cellulose and inhibit the water swelling property of cellulose to prevent its rupture. The regenerated lignin and micro/nanofibers interacted with each other through hydrogen bonding, while collaborating with each other using their own characteristics, thus achieving efficient oil–water separation.

#### Reusability, anti-oil fouling property and mechanical property

**Figure 6:**
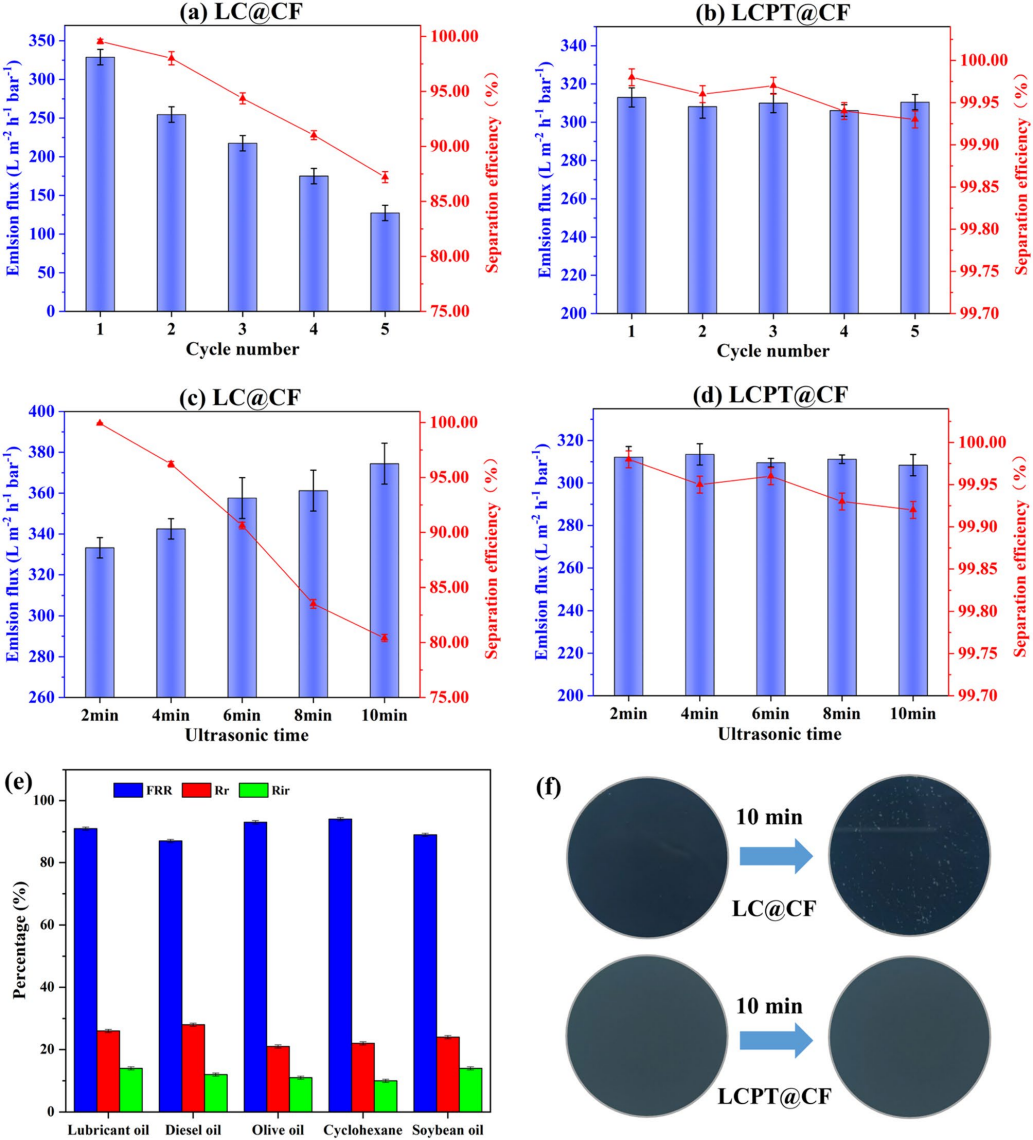
Emulsion flux and separation efficiency of (**a**) LC@CF and (**b**) LCPT@CF for multiple separations of the emulsions. Emulsion flux and separation efficiency of (**c**) LC@CF and (**d**) LCPT@CF after ultrasonic treatment for different times. (**e**) FRR, $R_{r}$ and $R_{ir}$ of LCPT@CF for separating different oil-in-water emulsions. (**f**) Photos of the LCPT@CF and LC@CF membranes after 10 min of ultrasonic.

The reusability of the membranes is also worth of attention regarding to the applications, and thus multiple recycling experiments of both LC@CF and LCPT@CF membranes were carried out. For the recycling experiments, olive oil-in-water emulsion was selected as an example, LC@CF and LCPT@CF membranes were washed by distilled water after each filtration, and the results were exhibited in Fig. 6a,b. The separation efficiency and flux of LC@CF gradually reduced as the separation cycle increased. After 5 cycles, the separation efficiency of LC@CF cut down from 99.92 to 87.41%, and the membrane flux of LC@CF reduced from 328.92 to 127.32 L/m^2^ h·bar, implying that the regenerated lignocellulose layer on the LC@CF membrane surface was easily destroyed and fell off during the filtration. In contrast, the separation efficiency of LCPT@CF remained 99.90% after five cycles. It could be concluded that the LCPT@CF possessed outstanding separation performance of emulsions due to the interactions of PT complexes and regenerated lignocellulose. These interactions further strengthened the bonding between the regenerated lignocellulose and the cotton fabric and improved the durability and overall mechanical properties of the LCPT@CF.

The anti-oil performance of LCPT@CF was also investigated for different types of oil-in-water emulsions. As shown in Fig. 6e, LCPT@CF had high FRR (> 85%), low $R_{r}$ (< 30%) and low $R_{ir}$ (< 15%) for different oil-in-water emulsions after five cycles. The results were demonstrated that LCPT@CF exhibited good resistance to oil pollutions due to the presence of a large number of hydroxyl groups on the surface of the regenerated lignocellulose, which can form a solid hydration layer on the surface of the LCPT@CF membrane.

Toward detecting the mechanical performance of LC@CF and LCPT@CF membranes during oil–water separation, the membranes were placed in ultrasonic water bath for 10 min at ambient temperature (Fig. 6f), and then LC@CF and LCPT@CF membranes were used to separate the emulsion. As illustrated in Fig. 6c,d, the separation efficiency of LC@CF decreased with the increase of ultrasonic time, and the membrane flux was opposite, which was attributed to the shedding of regenerated lignocellulose on the LC@CF membrane surface after ultrasonic treatment. By contrast, both the separation efficiency and flux of LCPT@CF did not change obviously, and all the separation efficiency was over 99.90%.

Furthermore, the comparison of the mechanical properties between LCPT@CF and original cotton fabric was conducted (Fig. S3). The enhanced mechanical properties of LCPT@CF were primarily due to the interfacial bonding between regenerated lignocellulose and the PT complexes with cotton fabric. The lignin, as a natural adhesive, enhanced the tight connection between cotton fibers, which improved the tensile strength of the LCPT@CF membrane. Additionally, the highly viscous PT complexes improved the overall integrity of the membrane, leading to a significant increase in its fracture elongation. The small amount of PVA on the surface of the LCPT@CF membrane also contributed to its mechanical strength. Moreover, the combination of lignocellulose and the PT complexes via hydrogen bonds further strengthened the overall integrity of the membrane. Therefore, the mechanical properties of LCPT@CF were significantly enhanced.

### Performance of different regenerated lignocellulose content

**Figure 7:**
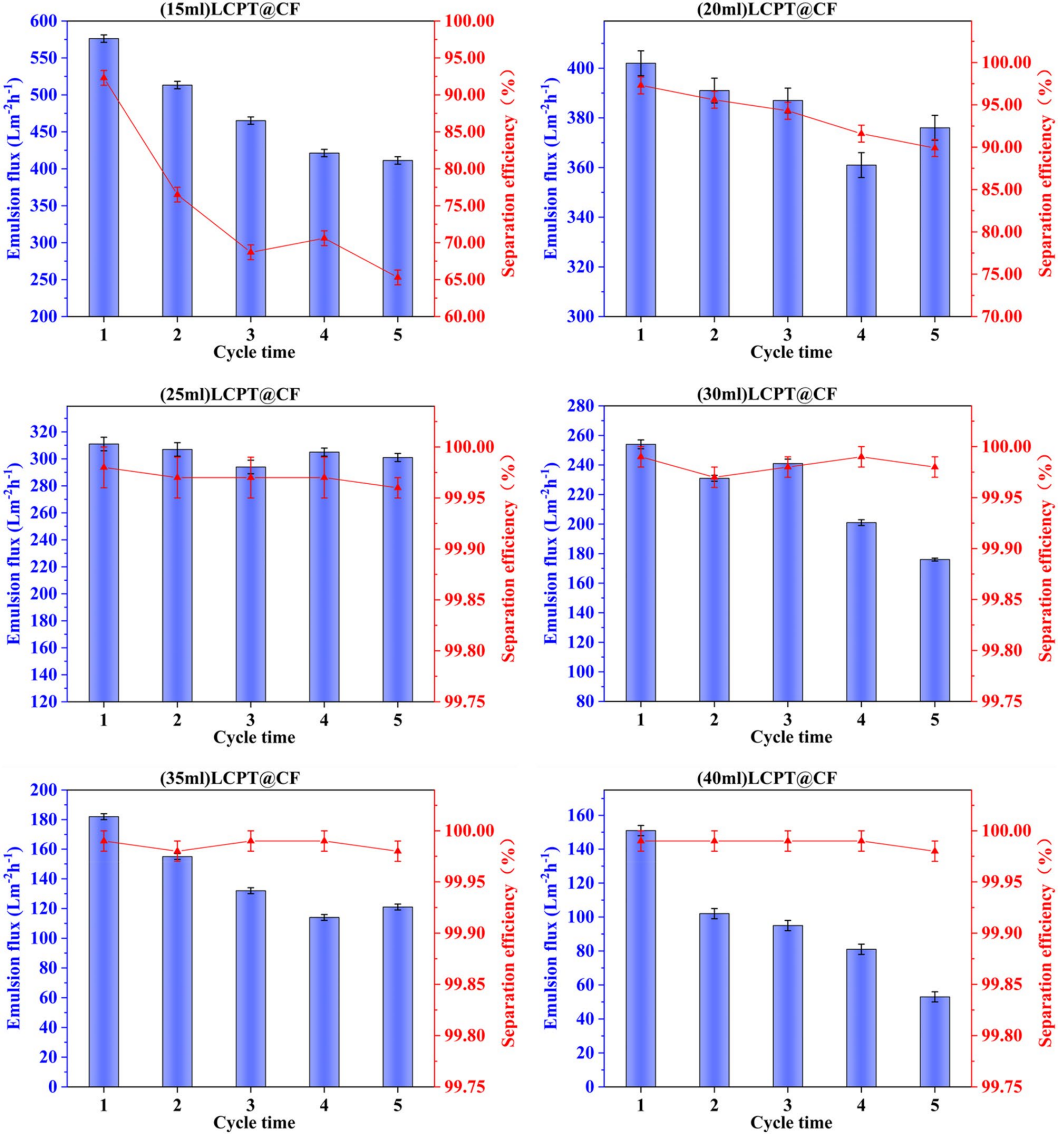
Emulsion flux and separation efficiency of LCPT@CF membranes with different contents of regenerated lignocellulose.

During the fabrication of LCPT@CF, the content of the regenerated lignocellulose solution ranged from 15 to 40 ml, and the performances of the LCPT@CF with different content of regenerated lignocellulose were investigated. As shown in Fig. 7, the separation efficiency of LCPT@CF could be enhanced with the increase of the content of regenerated lignocellulose, but its membrane flux decreased. With the increase of the regenerated lignocellulose content, the thickness of the LCPT@CF increased, which could increase the number of layers in the multilayered structure of the regenerated lignocellulose, thus enhancing the separation efficiency of the membrane. And most remarkably, the separation efficiency of the LCPT@CF prepared with 15–20 ml of the regenerated lignocellulose solution gradually decreased with the increase of the number of separation cycles, and its membrane flux gradually increased, which was ascribed to that the thin lignocellulose layer could be easily destroyed. When the lignocellulose solution content was in the range of 35–40 ml, the separation efficiencies of the LCPT@CF exceeded 99.95% after each the separation cycle.

### Solar evaporation tests

**Figure 8:**
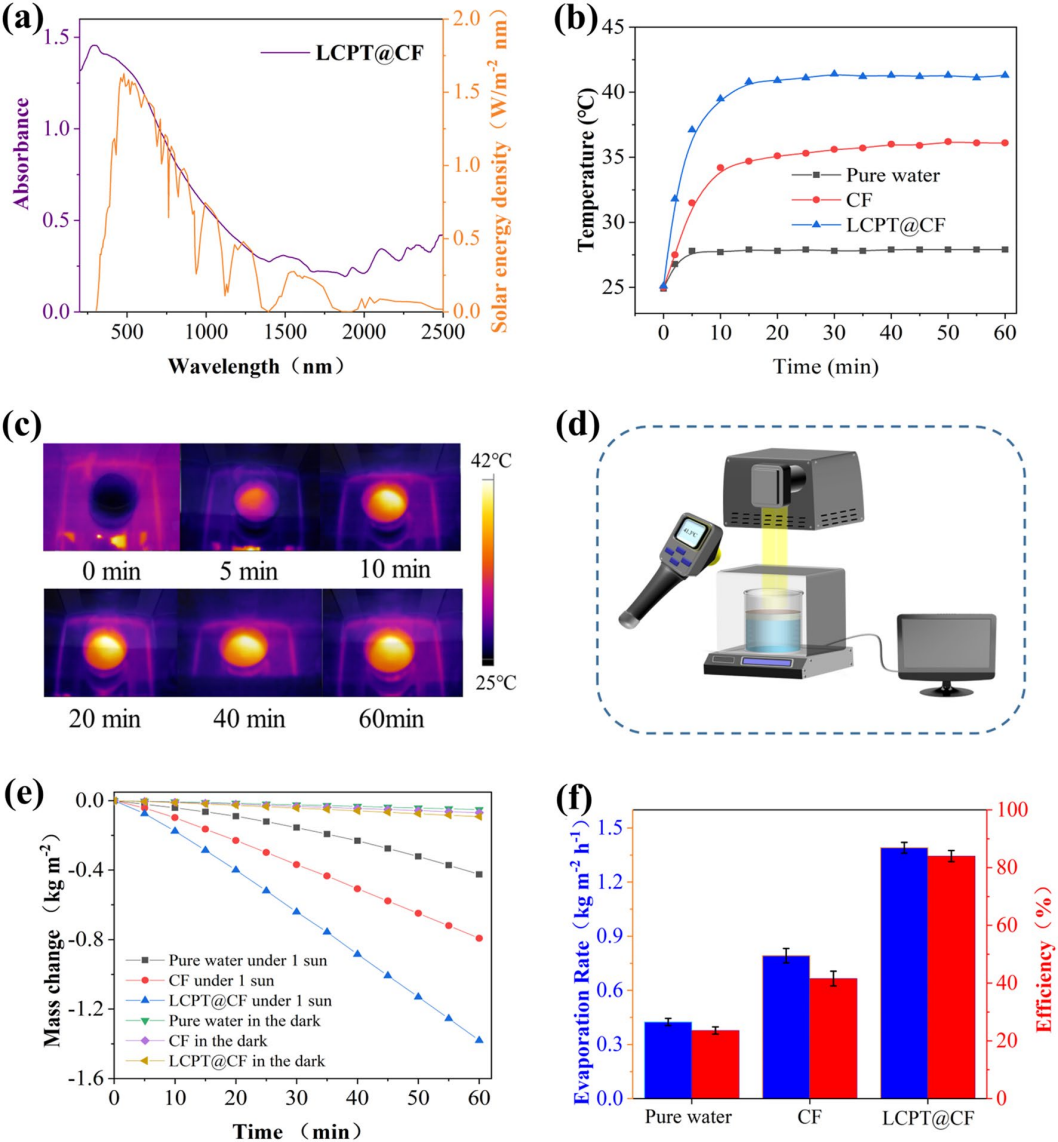
(**a**) Light absorption spectrum of LCPT@CF. (**b**) Surface temperatures of pure water, CF, and LCPT@CF. (**c**) IR photos of LCPT@CF. (**d**) Working mechanism of SSG system. (**e**) Mass changes of water over time in the dark environment and under 1 sun illumination. (**f**) Evaporation rates and efficiencies of pure water, CF, and LCPT@CF, respectively.

Light absorption capacity is a key factor in the photothermal conversion process. Figure 8a showed that the absorbance of LCPT@CF was across from 200 to 2500 nm. Obviously, the absorbance of LCPT@CF almost completely overlapped with the solar spectrum, which demonstrated that lignin was an ideal photothermal conversion material. The light absorption ability of the sample could also be reflected by the changes of surface temperatures and temperature distributions, which were showed in Fig. 8b and c. The surface temperatures of pure water and CF only increased 2.9 °C and 6.5 °C within 5 min, respectively. Whereas the surface temperatures of LCPT@CF rapidly increased by 12.1 °C, indicating the excellent thermal response of lignin. LCPT@CF demonstrated a significantly higher final surface temperature (41.3 °C) after one hour compared to pure water (27.9 °C) and CF (36.1 °C), respectively, indicating its efficient photothermal conversion capacity. This is attributed to the inherent π–π stacking of lignin molecules, which facilitates non-radiative transfer and triggers the photothermal conversion^23,24^. Specifically, the unique π–π stacking of lignin accounts for the significant difference of temperature, further confirming the remarkable solar-to-thermal conversion efficiency of LCPT@CF.

To comprehensively evaluate the evaporation performance of evaporator, the mass change of water was monitored via an electronic balance over time. The working mechanism of SSG system was showed in Fig. 8d. Under 1 sun, the evaporation rates of pure water and CF were 0.42 kg m^−2^ h^−1^ and 0.79 kg m^−2^ h^−1^, respectively (Fig. 8e). On the contrary, the evaporation rate of LCPT@CF was 1.39 kg m^−2^ h^−1^, which was 3.3 times faster than that of pure water alone. Notably, the evaporation rates of the samples contained the intrinsic evaporation rates in the absence of light, which were measured under dark condition. The intrinsic evaporation rates of water, CF, and LCPT@CF were 0.051, 0.069 and 0.091 kg m^−2^ h^−1^, respectively. The evaporation efficiency was calculated by using Eq. (7). As illustrated in Fig. 8f, the evaporation efficiency of LCPT@CF could reach as high as 84%, which was obviously superior to that of pure water (23.6%) and CF (41.6%).

### Salt resistance of lignocellulose-PVA-TA @ cotton fabric membrane

Effective evaporation performance of solar-powered evaporators is a critical factor in freshwater production, but salt tolerance is also a key consideration. Salt accumulation on the evaporator's surface can negatively impact steam escape rate and sunlight absorption capacity, leading to reduced photothermal conversion and evaporation performance. Therefore, salt tolerance is a crucial factor that can significantly affect the efficiency of solar-powered evaporators.

**Figure 9:**
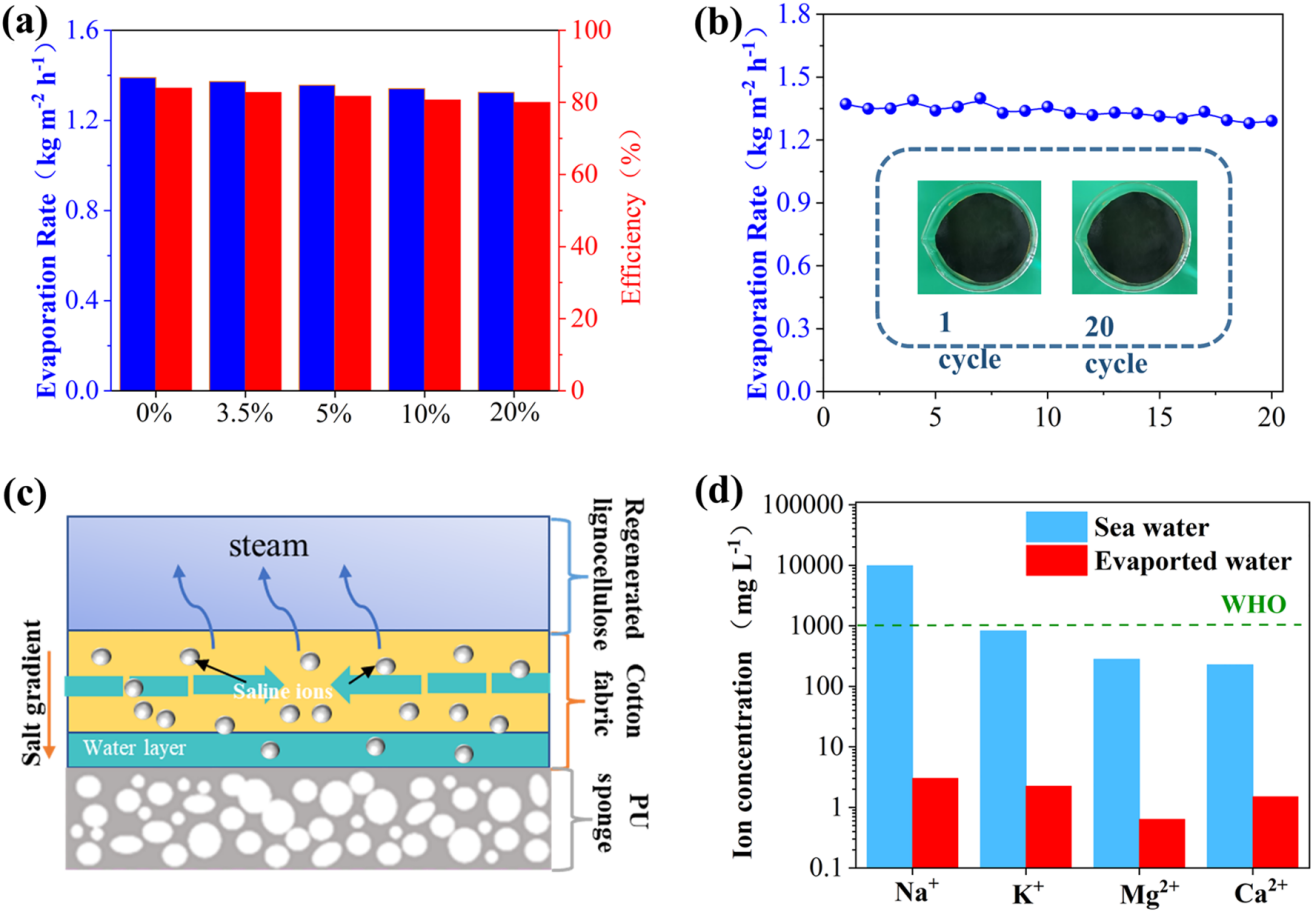
(**a**) Evaporation rates and efficiencies of LCPT@CF in saline water with different salt concentrations under one sun illumination. (**b**) Cycling performance of LCPT@CF in seawater under one sun illumination (the inset is the photograph of LCPT@CF before and after 20 cycles). (**c**) Schematic of the transport path of salt ions (**d**) Concentrations of Na^+^, K^+^, Mg^2+^, Ca^2+^ in seawater and evaporated water.

The salt resistance performance of the LCPT@CF was investigated by evaporating different concentrations of saline water. When the brine concentration was further increased to 20 wt%, the corresponding efficiency was still above 80% (Fig. 9a). In addition, to evaluate the stability of LCPT@CF in practical application, seawater obtained from East China Sea near Zhoushan city was used to measure its practical evaporation performance. Figure 9b showed that the LCPT@CF could maintain steady evaporation rates during 20 working cycles. The hydrophobicity of lignin prevents salt deposition on the membrane's surface, which was shown in the inset of Fig. 9b. Due to the deposited lignin, concentrated saline water cannot reach the cotton fabric surface, and evaporation only occurs at the hydrophobic and hydrophilic interfaces. Salt crystals dissolve in the hydrophilic layer and bulk water, rather than aggregating on the hydrophobic surface (Fig. 9c). The rapid water exchange in the hydrophilic layer prevents salt deposition, which was confirmed in the Fig. 9b inset. Therefore, these findings demonstrate that the hydrophobicity of lignin plays a crucial role in preventing salt accumulation on the membrane's surface and maintaining its effectiveness in evaporation^46^. Interestingly, it was revealed that the concentrations of four main metal ions in including Na^+^, Mg^2+^, K^+^, Ca^2+^ were decreased to 1.72, 1.34, 0.47, and 0.96 mg L^−1^, respectively (Fig. 9d), which were fully meet the standards of drinking water by the World Health Organization (WHO)^47^. These results demonstrated that LCPT@CF had efficient and practical ability for desalination.

### Degradability of lignocellulose-PVA-TA @ cotton fabric membrane

**Figure 10:**
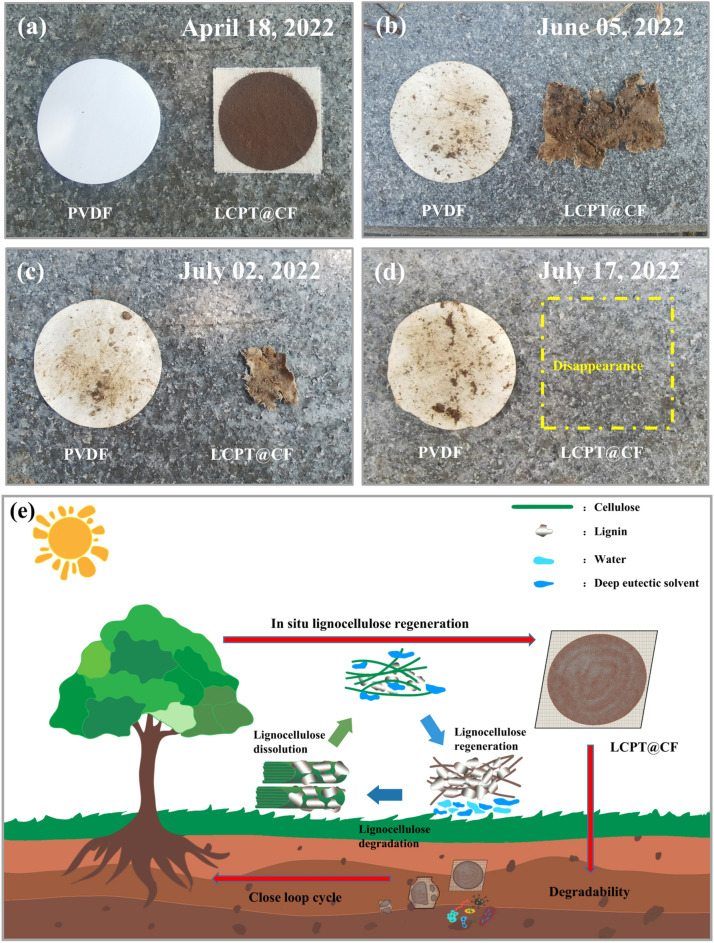
(**a**–**d**) Degradation experiments of PVDF and LCPT@CF membranes under natural soil burial conditions. (**e**) Schematic diagram of the closed-loop cycle of LCPT@CF.

The biodegradable properties of LCPT@CF were studied under natural soil burial condition. For comparison, LCPT@CF and polyvinylidene fluoride (PVDF) membrane were buried in soil at a depth of 10 cm and their morphologies were monitored on the degradability over time (Fig. 10a–d). Microorganisms can directly digest macromolecules of cellulose and lignin in the LCPT@CF membrane. Thus, the regenerated lignocellulose was gradually degraded by microorganisms after 3 months. Additionally, PVA and TA can be used by microorganisms as carbon and energy source^48^. Therefore, there is no pollution for LCPT@CF on the environment during the process of degradation. Satisfactorily, the LCPT@CF was completely degraded by microorganisms after 3 months in soil. The degradation products of LCPT@CF can be absorbed by trees, thus forming a closed loop (Fig. 10e). However, the PVDF membrane kept the original shape without any change in the same condition. Consequently, we were able to infer the degradability and environmental friendliness of the LCPT@CF.

## Conclusion

In summary, we demonstrated a simple and economical method to prepare a green multifunctional membrane for oil–water separation and seawater desalination by in situ regeneration of lignocellulose. The regenerated lignocellulose was bonded with cotton by PT complexes, which could be fully utilized to purify water. The results confirmed that LCPT@CF possessed outstanding separation efficiency (99.90%) and reasonable membrane flux (313 L/m^2^ h·bar) for oil-in-water emulsions, as well as excellent anti-fouling properties and environmental friendliness. Meanwhile, the LCPT@CF could separate a variety of oil-in-water emulsions. Additionally, the solar vapor generation efficiency of LCPT@CF is 84%, and the evaporation rate is 1.39 kg m^−2^ h^−1^ under one sun irradiation. Therefore, the preparation of multifunctional membrane of LCPT@CF by the regenerated lignocellulose not only broadened the utilization of lignin, but also provided a new solution for oil–water separation and seawater desalination. Furthermore, the original materials for the preparation of LCPT@CF had excellent environmental friendliness and could be degraded by natural microorganisms in the natural condition within 3 months. Therefore, the green and pollution-free LCPT@CF exhibited potential application in the treatment of oil–water separation and seawater desalination, and this study provided a new route to prepare green, stable and biodegradable multifunctional membrane for oil–water separation and seawater desalination.

## Supplementary Information

Supplementary Information 1.

Supplementary Video 1.

Supplementary Video 2.

## Data Availability

The data is available from the corresponding author on reasonable request.
