# Supplementary material for: Environmental-friendly regenerated lignocellulose functionalized cotton fabric to prepare multi-functional degradable membrane for efficient oil–water separation and solar seawater desalination
Source: Sci Rep. 2023 Mar 31;13:5251. doi: 10.1038/s41598-023-32566-9 (PMC10066188; doi:10.1038/s41598-023-32566-9)
Supplement: Supplementary file 1 — Supplementary Information 1. [file 41598_2023_32566_MOESM1_ESM.docx]

***Supporting Information for***

**Environmental-friendly regenerated lignocellulose functionalized cotton fabric to prepare multi-functional degradable membrane for efficient oil-water separation and solar seawater desalination**

Jiangyi Li^1^, Junkai Gao^1^, Jiangyu Fang^1^, Tian Ling^1^, Mengsheng Xia^1^, Xue Cao^1^, Zhi Han^2^, Yan Chen^1*^ (*Corresponding author. Tel.: +86 580 2550623. E-mail: chenyan@zjou.edu.cn)

^1^ School of Naval Architecture and Maritime, Zhejiang Ocean University, Zhoushan 316022, China.

^2^ School of Energy and Power Engineering, Jiangsu University, Zhenjiang 212013, China


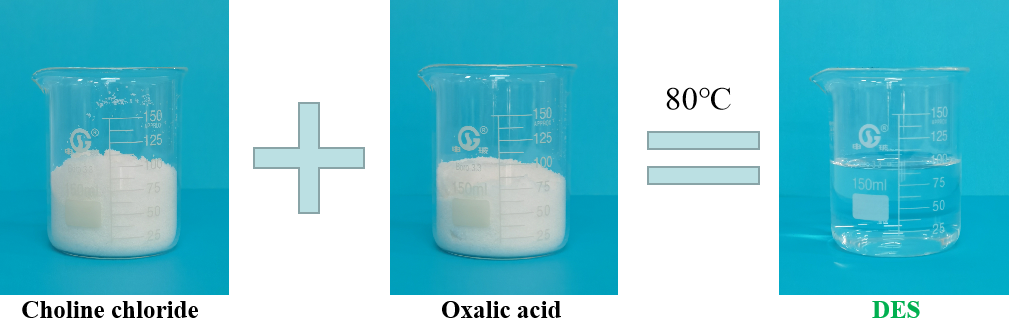


**Fig.S1** The preparation process of DES

**Table S1.** Elemental compositions of membrane surface

| Membranes | Composition (%) | | | Element ratio |
| --- | --- | --- | --- | --- |
|  | C | O | N | O/C |
| CF | 74.57 | 25.06 | 0.37 | 0.34 |
| LC@CF | 65.29 | 34.47 | 0.24 | 0.53 |
| LCPT@CF | 62.35 | 37.09 | 0.56 | 0.59 |


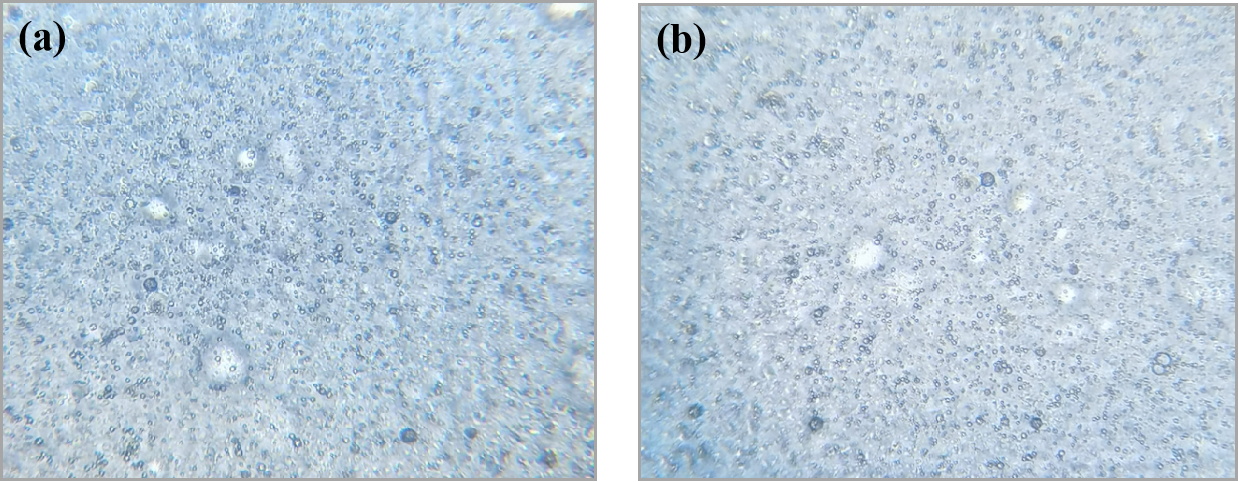


**Fig.S2** (a) Biomicroscope image of olive oil-in-water emulsion. (b) Biomicroscope image of olive oil-in-water emulsion after 48 hours.


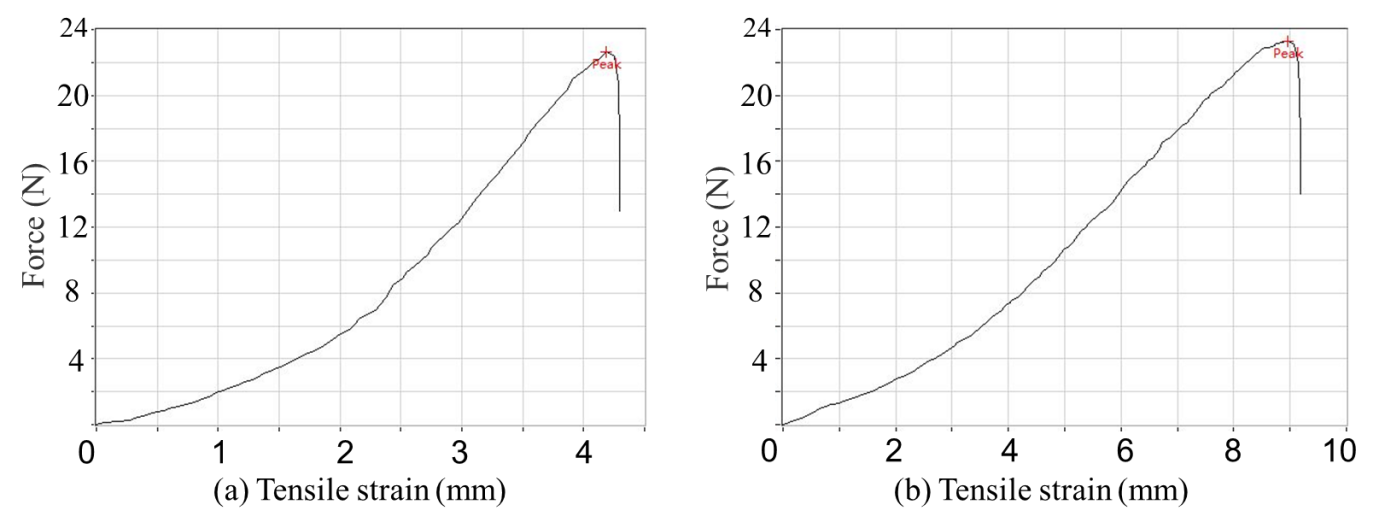


**Fig.S3** (a) Mechanical property of original cotton fabric. (b) Mechanical property of LCPT@CF.

**The comparison of surface morphology of LCPT@CF before and after desalination and oil-water separation**

As shown in Fig. S4 a1and b1, the surface morphology of LCPT@CF membrane remains unchanged before and after oil-water separation. Meanwhile, as observed in Fig. S4 d1, there is no significant change in the C 1s and O 1s peaks of LCPT@CF membrane after oil-water separation, indicating that there is no oil deposition on the membrane surface. After seawater desalination, in the figure S4 a1and c1, no salt particles were found on the membrane surface of LCPT@CF, indicating excellent salt resistance. As shown in Fig.S4 d2, the C 1s and O 1s peaks of LCPT@CF did not obvious change after seawater desalination, indicating high stability of the membrane during the desalination process. These results demonstrated that LCPT@CF had excellent oil-pollution resistance and outstanding seawater desalination ability.


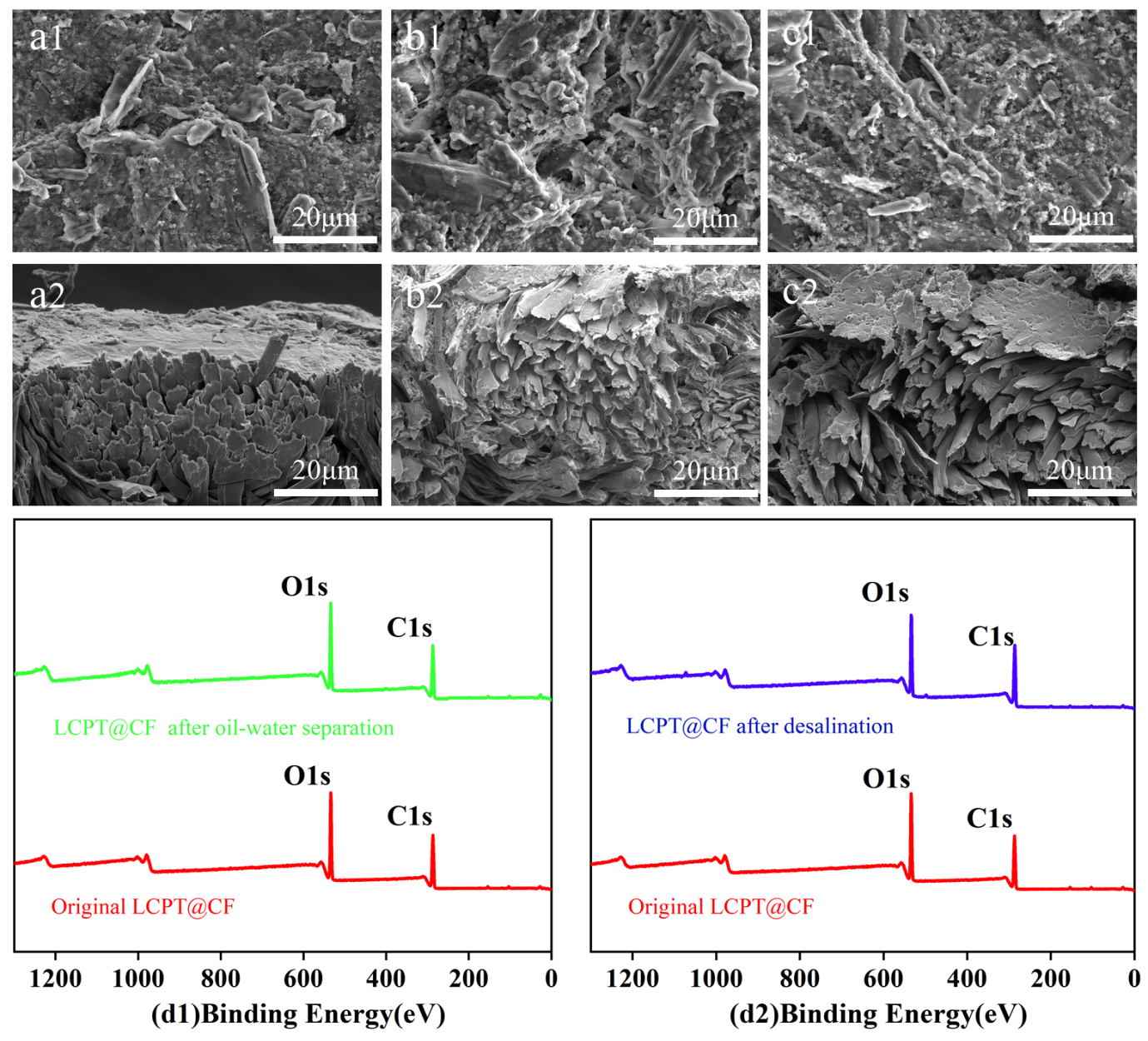


**Fig.S4** (a1-a2) Top-view SEM image and cross-sectional SEM image of original LCPT@CF. (b1-b2) Top-view SEM image and cross-sectional SEM image of LCPT@CF after oil-water separation. (c1-c2) Top-view SEM image and cross-sectional SEM image of LCPT@CF after desalination. (d1) XPS Spectrum of LCPT@CF before and after oil-water separation. (d2) XPS Spectrum of LCPT@CF before and after desalination.

**The Comparison of LCPT@CF with other materials for oil-water separation and seawater desalination**

As shown in Table S2, LCPT@CF and other cotton fabrics used for emulsion separation were compared in terms of separation efficiency, material properties, and environmental hazards. The results demonstrated that LCPT@CF exhibited excellent separation efficiency for oil-in-water emulsions. In addition, as shown in Table S3, LCPT@CF was compared with other materials for seawater desalination in terms of evaporation rate, evaporation efficiency and durability. The results showed that LCPT@CF exhibited excellent separation efficiency for seawater desalination. Moreover, LCPT@CF was environmentally friendly, as the combination of lignocellulose and PT did not cause pollution to the environment. Additionally, the preparation of LCPT@CF was simple and cost-effective. Therefore, LCPT@CF had enormous potential for practical applications in oil-water separation and seawater desalination.

**Table S2.** Comparison of LCPT@CF and other cotton fabric membranes used for the emulsion separation

| Membranes | Coating material | Environmental hazard | Separation  efficiency (%) | Ref. |
| --- | --- | --- | --- | --- |
| Mg(OH)_2_@cotton fabric | Mg(OH)_2_ | Middle | >99.5 | ^1^ |
| Caffeic acid/Fe@cotton fabric | Caffeic acid/Fe | Middle | >99.0 | ^2^ |
| Robustly superhydrophobic  cotton fibers | ZnO | Middle | >91.0 | ^3^ |
| Eleostearic acid-SiO_2_@cotton fabric | SiO_2_ and eleostearic acid | Middle | >99.5 | ^4^ |
| CS@CF | Cement-sand | Small | >97.2 | ^5^ |
| Cellulose-coated  cotton fabric | Cellulose | Small | >93.2 | ^6^ |
| Superwetting cellulose II based membranes | Cellulose | Small | >99.0 | ^7^ |
| LCPT@CF | Regenerated lignocellulose | None | >99.9 | **This work** |

**Table S3**. Performance of LCPT@CF compared to other evaporators

| Evaporator | Evaporation rate  (kg m^-2^ h^-1^) | Evaporation  efficiency (%) | Durability | Ref. |
| --- | --- | --- | --- | --- |
| Carbonized mushrooms | 1.12 | 78 | 8 cycles | ^8^ |
| Modified coated cotton | 1.19 | 80 | 10 cycles | ^9^ |
| JCA | 1.81 | 92.5 | 10 h | ^10^ |
| AC-BS | 1.51 | 86.8 | 20 cycles | ^11^ |
| LCPT@CF | 1.39 | 84 | 20 cycles | **This work** |

# References

1. Hu, R. et al. Fabrication of special wettability functionalized Mg(OH)2@cotton fabric for oil/water mixtures and emulsions separation. Cellulose 27, 7739–7749 (2020).

2. Zhou, Q., Yan, B., Xing, T. & Chen, G. Fabrication of superhydrophobic caffeic acid/Fe@cotton fabric and its oil-water separation performance. Carbohydrate Polymers 203, 1–9 (2019).

3. Wang, J., Han, F., Liang, B. & Geng, G. Hydrothermal fabrication of robustly superhydrophobic cotton fibers for efficient separation of oil/water mixtures and oil-in-water emulsions. Journal of Industrial and Engineering Chemistry 54, 174–183 (2017).

4. Lin, H. et al. Facile fabrication of natural superhydrophobic eleostearic acid-SiO2@cotton fabric for efficient separation of oil/water mixtures and emulsions. Sustainable Materials and Technologies 32, e00418 (2022).

5. Yang, S., Li, M., Fang, G., Xue, M. & Lu, Y. Flexible cement-sand coated cotton fabrics with superhydrophilic and underwater superoleophobic wettability for the separation of water/oil mixtures and oil-in-water emulsions. Colloids and Surfaces A: Physicochemical and Engineering Aspects 608, 125611 (2021).

6. Zhang, Y.-R., Chen, J.-T., Hao, B., Wang, R. & Ma, P.-C. Preparation of cellulose-coated cotton fabric and its application for the separation of emulsified oil in water. Carbohydrate Polymers 240, 116318 (2020).

7. Yang, S., Chen, L., Wang, S. & Liu, S. Production of superwetting cellulose II based membranes with excellent oil/water emulsions separation performance. Industrial Crops and Products 177, 114554 (2022).

8. Xu, N. et al. Mushrooms as Efficient Solar Steam-Generation Devices. Adv. Mater. 29, 1606762 (2017).

9. Wilson, H. M., Rahman A.R., S., Parab, A. E. & Jha, N. Ultra-low cost cotton based solar evaporation device for seawater desalination and waste water purification to produce drinkable water. Desalination 456, 85–96 (2019).

10. Alam, Md. K. et al. Stable and Salt-Resistant Janus Evaporator Based on Cellulose Composite Aerogels from Waste Cotton Fabric. ACS Appl. Mater. Interfaces 14, 41114–41121 (2022).

11. Chen, Y. et al. Bamboo shoot-based evaporator with self-cleaning and mildew-resistant for efficient solar steam generation. Desalination 541, 116003 (2022).
